# Public Health Workforce Professional Development Analysis: A Human-Systems Integration for Healthy Communities

**DOI:** 10.3389/ijph.2025.1608006

**Published:** 2025-02-27

**Authors:** Bashkin Osnat, Baron-Epel Orna, Bochenek Tomasz, Czabanowska Kasia, Davidovitch Nadav, De Nooijer Jascha, Dopelt Keren, Duplaga Mariusz, Harrington Janas, Leighton Lore, Levine Hagai, MacLeod Fiona, Malowany Maureen, Mor Zohar, Neumark Yehuda, Okenwa-Emegwa Leah, Otok Robert, Paillard-Borg Stephanie, Peled-Raz Maya, Tulchinsky Theodore, Zelber-Sagi Shira, Barach Paul

**Affiliations:** ^1^ Department of Public Health, Ashkelon Academic College, Ashkelon, Israel; ^2^ School of Public Health, Faculty of Social Welfare and Health Sciences, University of Haifa, Haifa, Israel; ^3^ Department of Health Promotion and e-Health, Institute of Public Health, Faculty of Health Sciences, Jagiellonian University Medical College, Kraków, Poland; ^4^ School of Health Professions Education, Faculty of Health, Medicine and Life Sciences, Maastricht University, Maastricht, Netherlands; ^5^ Department of Health Policy and Management, School of Public Health, Faculty of Health Sciences, Ben Gurion University of the Negev, Beer Sheva, Israel; ^6^ The Israeli Association of Public Health Physicians (IAPHP), Israeli Medical Association, Ramat Gan, Israel; ^7^ The Association of Schools of Public Health in the European Region (ASPHER), Brussels, Belgium; ^8^ School of Public Health, University College Cork, Cork, Ireland; ^9^ Braun School of Public Health and Community Medicine, Hebrew University of Jerusalem, Jerusalem, Israel; ^10^ Department of Health Sciences, The Swedish Red Cross University, Huddinge, Sweden; ^11^ College of Population Health, Thomas Jefferson University, Philadelphia, PA, United States; ^12^ College of Population Health, Sigmund Freud University, Vienna, Austria; ^13^ Sheps Center for Health Services Research, University of North Carolina, Chapel Hill, NC, United States

**Keywords:** human factors, public health workforce development, training, organizational factors, education

## Abstract

**Objectives:**

The healthcare landscape is challenged by emerging and severe public health threats, and fast shifting priorities. There is an urgent need to build public health workforce capacity to enable rapid adaptation and effective responses to these threats. We outline a whole system’s learning approach for analyzing public health systems in collaboration with public health leaders.

**Methods:**

The project included: i) a system’s analysis involving a cross-sectional mixed methods approach including a quantitative investigation, interviews and focus groups of leading representatives and students from five higher education institutions providing public health training, 49 managers, and 31 stakeholders from lead public health organizations; ii) develop and implement training interventions, involving human factors tools for evaluating and developing actionable solutions.

**Results:**

We developed and implemented three interventions: 1) An online user interface for public health professional development and collaboration; 2) A Public Health Leadership Academy; and 3) A video outreach to key stakeholders and communities using studies.

**Conclusion:**

A holistic perspective incorporating human factors, and a systems approach provided a comprehensive understanding and approch towards the public health workforce while identifying leverage points for durable improvement.

## Introduction

Public health systems face complex challenges that require structural flexibility and constant adaptations to facilitate innovative and agile capacity-building solutions that address increasing global health inequities. There considerable gaps between current public health (PH) evidence-based recommendations and what is delivered to populations [1]. The effectiveness of PH systems in a rapidly changing world relies on an infrastructure of competent, multidisciplinary workforce microsystems involved with designing and implementing PH interventions to meet emerging and existential challenges [2]. Developing and improving the PH workforce competencies requires a deep as well as comprehensive and nuanced understanding of the complex nature of the community-focused PH system challenges using whole systems thinking. We hypothesized that a novel framework for analyzing and improving the organizational, training, and educational factors is needed to enhance the professional development and sustainment of the PH workforce of the future.

### The Public Health System

The PH system is shaped by multiple influences, interconnections, and feedback loops [3]. Considering growing inequalities and evidence of variable quality, there is a growing interest in applying health systems-based perspectives to public health resilience [4]. We define resilience in public health as the ability of communities and health systems to adapt to, recover from, and withstand public health emergencies and disasters. It’s important for improving population health and wellbeing. Systems-based approaches involve mapping the relationships among multiple factors that influence health outcomes and using them to identify potential points of intervention, working with key stakeholders to understand the context, and developing community-focused interventions, that consider the distinctive characteristics of complex systems [5]. System-based approaches incorporate interdisciplinary research methods and tools analyzing processes, human-system interactions, and key contextual factors using mixed-methods models and participatory-based research [6, 7].

A recent systematic review found that adopting systems-based approaches was associated with improved patient and service outcomes [8]. Several factors contributed to these benefits, including better stakeholder engagement, communication enhancement, team-based and collaborative approaches.

In a learning PH system, internal data and experience are systematically integrated with external evidence, and knowledge is put into daily practice [9]. As a result, citizens get higher quality, safer, more efficient, and more responsive PH services, and PH delivery organizations become better workplaces.

### Human Factors Perspective in Public Health

One of the better known system-based approaches is the human factors approach. It is defined as the analysis and design of the systems within which people work, whether those systems are technologies, processes, or organizations [10]. The study of human factors is based on a sociotechnical view that focuses on human performance and interactions with equipment, systems, and processes within the organization to enhance performance, increase safety, and improve user satisfaction [11, 12]. The human factors approach in health adopts a whole system’s thinking that aims to identify related vulnerable societal points and treat these points throughout the work environment, addressing blocks in care, bridging the gaps between human capabilities and work requirements, and redesigning health systems to better serve patients and the staff that care for them—always assessed through the lens of real-world conditions [13, 14].

Human-centered design, a cornerstone of the human factors approach, uses multiple user-centered co-design, active learning, and feedback loops [15]. This iterative process, first pioneered by Edwards Deming’s Theory of Profound Knowledge and articulated in his 14 principles of management, starts with creating a *“*constancy of purpose” toward the improvement of public health services and must be built around honest collaboration within the organizational learning system and co-produced with community partners, community demands and resources included in the design of PH services [16]. As PH design and implementation take shape, there is a need to build tools carefully designed, contextually appropriate, and multifaceted to motivate citizens to create and sustain an effective learning system that works for, and in, their communities [9].

### The Israeli Public Health System

The structure of health services in Israel combines mandatory state insurance with additional supplementary non-profit healthcare plans that promote health and longevity and prevent diseases. Every citizen or permanent resident of Israel can choose from four competing nationwide Health Maintenance Organizations (HMOs) that must provide their members access to a statutory benefits package. The Ministry of Health in Israel is responsible for the regulation of the health system and facilitates PH services. It provides national leadership in a broad range of PH domains, including food safety, control of communicable and non-communicable diseases, screening, health promotion, environmental health, and epidemiological monitoring. The structure of the PH system includes the headquarters’ units responsible for policymaking and issuing guidelines to regional health departments that provide community-based services and operate mother and child health clinics. The regional departments are operated by physicians with PH expertise, public health nurses, environmental epidemiologists, and other public health-related professionals.

Israel’s PH challenges have become more urgent and complex. Israel’s population maintained its high growth rates (1.93% average annual growth rate), and at the end of 2023, the population in Israel was 9.84 million [17]. Longevity has also increased, and while global life expectancy at birth in 2022 was 72 years, 80 years in OECD countries and 77 years in the United States, the average life expectancy at birth in Israel was 83 years [18]. However, Israel faces challenges such as rising poverty and growing population inequities [19, 20]. Emerging challenges include state security, climate change [21], food insecurity [22, 23], ensuring a safety net for uninsured migrants [24], providing health services during war and conflicts [25] and emerging and re-emerging Infectious diseases such as COVID-19, mpox, and vector-borne diseases [26–28], while non-communicable diseases have moved to the forefront of an aging population in the form of cardiovascular disease, diabetes, obesity, and cancer [29]. In addition, new digital, organizational, and scientific advances bring opportunities but also unintentional consequences for the welfare of the state’s citizens such as overload anxiety, and addiction [30].

This paper describes the research questions and experimental findings that framed the multinational Erasmus Plus project for building Capacity in Higher Education entitled “Sharing European Educational Experience in Public Health for Israel (SEEEPHI) [31]: harmonization, employability, leadership, and outreach”. The key purpose of the project was to apply a whole system’s learning and an evidence-based approach to analyze the PH system in Israel. This work was done in close cooperation with European academic partners to reflect on the shared experiences in addressing challenges with upskilling education and training of the PH workforce in addressing the needs of employers, and mindful of the broader social environment across varied regional contexts, academic and occupational governance needs [31].

## Methods

### SEEEPHI Project Background

The project brought together the main consortium of PH schools in Europe and Israel-ASPHER (Association of Schools of Public Health in the European region). Four Israeli academic institutions provide Master in PH program (MPH) training programs: The Ben Gurion University of the Negev (BGU), The Hebrew University of Jerusalem (HUJI), the University of Haifa (UOH), and Ashkelon Academic College (AAC) which provides an undergraduate PH program. In addition, the Israeli Association of Public Health Physicians (IAPHP), a professional society in the Israeli Medical Association, (the official representative of Israel in EUPHA and WFPHA), was part of the consortium. The European higher education institutions (HEI) partners included: Jagiellonian University Medical College of Kraków, Poland; University College Cork, Ireland; Maastricht University, the Netherlands; and the Swedish Red Cross University, Sweden. In addition, the Association of Schools of Public Health in the European Region (ASPHER) coordinated the project.

### Study Design

The project was conducted between January 2021 and September 2024. It included two phases: 1) system analysis; and 2) the design and implementation of interventions, applying mixed-methods systems-based research. The data collection protocols were approved by the ethics committees of the participating Israeli higher education institutions and written consent was obtained when needed (AAC, BGU, UOH, HUJI).

A detailed system analysis of field qualifications was performed to understand the Israeli PH system’s different professional roles and required competencies. In parallel, mapping the profiles of the PH competencies of the 5 Israeli PH programs was conducted to initiate harmonization in teaching modalities and training programs.

Design and implementation of interventions and teaching programs were based on data collected in the analysis phase 1 and the evaluation of these solutions, including: 1) Development and implementation of a dynamic online interface to enable PH training–practice collaborations, supporting employability and continuing professional development in the Israeli PH system; 2) Building leadership training programs in the Israeli PH schools and programs; and 3) Stakeholder engagement to secure outreach (community, inter-professional, cross-sectoral) needed to sustain the essentials of the program.

The SEEPHI project research questions were:

1. What are the current and anticipated competency gaps between public health training programs and workforce needs in Israel?
2. How well do existing public health academic programs in Israel cover core public health competencies as defined by international standards?
3. What are the perceived gaps between academic training and professional practice among public health graduates?
4. How effective are simulation-based and problem-based learning approaches in developing public health leadership competencies?
5. What interventions can effectively bridge the identified gaps between public health education and practice?

### Participants

The key participants in the SEEEPHI project included:

1. Higher Education Programs of PH: representatives of 5 Higher education PH programs in Israel participated in the project. One institute offers an undergraduate program (AAC), and four institutions offer an MPH program. In addition, utilizing the convenience sampling method, 18 students studying for a master’s degree in health policy and management at the Ben Gurion University School of Public Health participated in the project, 56% were women, with ages ranging from 26 to 50 (average 38 ± 7.23); 4 (22%) doctors, 5 (28%) nurses, 4 (22%) health professionals, 5 (28%) administrative positions [32].
2. MPH graduates: utilizing a census sampling method, 127 MPH degree graduates from UOH participated in the project 74.8% were women, and the mean age of participants was 40.7 years [33].
3. PH and Healthcare organizations: utilizing a purposive sampling method 49 managers (67.3% women) participated in the project, representing various organizations in Israel, such as hospitals, HMOs, NGOs, regional health departments, governmental offices, and research institutes [34].
4. PH key stakeholders: utilizing a purposive sampling method, 31 diverse stakeholders from PH organizations in Israel participated in the project, 67% women, 22 (71%) PH physicians (MD and MPH), 4 (13%) PH nurses (with MPH), 3 (10%) health promotion professionals, 2 (6%) environmental and food inspectors [35].

### Research Methods and Analysis

We applied mixed-methods research to collect and analyze data to implement and evaluate novel solutions. These methods included:

#### Quantitative Research

We conducted four types of surveys:

1) Survey mapping the workforce’s current and anticipated competencies [34] using an adapted version of the validated WHO-ASPHER Competency Framework for the Public Health Workforce in the European Region [36] (N = 49).
2) Survey of MPH alumni exploring their perceptions about personal competencies, job performance, and professional development [33] (N = 127).
3) Survey mapping the PH competencies included in the curricula of HEI providing PH education. This survey used an abridged version of the ASPHER list of Core Competencies for the Public Health Professional [37] to map PH competencies taught by HEI providing PH training [38] (N = 5).
4) Survey exploring students’ self-reported knowledge, skills, and gaps in their knowledge base through a questionnaire that comprised open- and closed-ended questions that explored students’ self-reported knowledge, skills, and gaps in their knowledge base [32] (N = 18).

#### Qualitative Research

1) In-depth video-recorded interviews were conducted with 31 key health professionals to identify the gaps between training programs and the competency demands of real-world challenges [35].
2) In-depth interviews were conducted with 16 out of 18 MPH students who participated in the simulations to examine simulations-based training impact and student satisfaction [32].
3) In-depth interviews with 24 MPH graduate alumni about their personal competencies, job performance, and professional development perceptions [33].

All interviews were conducted by research assistants, three Masters students trained in qualitative research methods and supervised by the SEEEPHI senior project staff.

Table 1 summarizes in detail the methods, data processing and analysis, and quality control applied in each of the phases.

**TABLE 1:** Research methods, data processing and analyses, and quality control of the SEEEPHI project by project phases (Israel, 2024).

Project Phase	Research Method	N	Methods Summary	Statistical Analysis	Quality control
Phase 1 - Detailed analysis of field qualifications content	Quantitative Qualitative	49 31	Online survey among Israeli PH managers to assess 44 competencies and core organizational PH operations Semi-structured interviews with mid to senior-level managers in the Israeli PH service, health funds, and hospitals	Calculated reliability using Cronbach’s alpha; used percentages to test differences among organizations; deficiency rates calculated for each competency Thematic analysis based on grounded theory [39] [2003]	Modified Delphi method for questionnaire design; conducted reliability testing; data analyzed consistently according to project protocol Conducted ongoing internal quality audit; standardized codebook for translation; pilot testing of the interview guide; internal quality audit [40]
Phase 1 - Mapping of competency profiles of schools and programs of PH	Quantitative	5	Representatives from Israeli Health Education Institutions responded to a survey tool consisting of 57 competencies in six domains	Relative coverage was ranked on a Likert scale; analyzed competencies addressed and their relative coverage	The survey tool was piloted and reviewed
Phase 1-Mapping of alumni competencies & satisfaction levels	Quantitative Qualitative	127 24	Explanatory sequential design Interviews transcribed and analyzed by two researchers	Descriptive statistics Thematic analysis of the transcribed data	The survey tool was piloted and reviewed Standardized codebook for translation; pilot testing of the interview guide
Phase 2 - Building leadership capacity	Quantitative Qualitative	18 16	Developed and piloted a leadership course using training simulations for health professions. Conducted pre-post surveys with students Developed and piloted a leadership course using training simulations for health professions. Semi-structured interviews with students	Frequency distribution for questionnaire responses Thematic analysis for interviews based on Kolb’s experiential learning model [41]	The survey tool was piloted and reviewed; used a standardized codebook Frequent meetings for consistency; internal quality audit [40]

### Ethics

The project was approved by the Ashkelon Academic College Ethics Committee (approval # 31-2021), by the ethics committee of Ben Gurion University of the Negev (approval #198-1 dated May 25, 2022), and by the ethics committee of the University of Haifa (approval #060/22). Informed consent was obtained from all individual participants included in the project.

## Results

### Phase 1: Data Analysis

The following sections describe the analysis stage of the project, including analyzing the field qualifications content (survey 1 followed by in-depth interviews), mapping alumni competencies & satisfaction levels (survey 2 followed by in-depth interviews), and, mapping the competency profiles of the PH schools and programs(survey 3).

#### Field Qualifications Map

We explored the compatibility between PH training programs and the practical competencies required by employers to address current and future PH needs in Israel. 49 Israeli PH managers completed an online survey from August to November 2021 [34]. In addition, 31 key health professionals were interviewed.

The assessment focused on identifying the gaps in the competencies required for effective PH practice. We sought to determine which of the training courses support the PH workers and how best to ensure that the training sessions are effective for the workers in the PH field. Mapping the currently required qualifications of the PH workforce indicated a pervasive deficiency of essential competencies, an unfulfilled need for better-trained workers, and a clear need to better integrate the PH workforce with the right competencies to deal with increasingly complex and diversified tasks. Table 2 describes the deficiencies reported by health organizations [34].

**TABLE 2:** Competency deficiencies as reported by organizations^a^ for the SEEEPHI project, % of respondents) (Israel, 2024).

Competency Domain	Organizations			
	Hospitals, HMOs, NGOs (N = 19)	Health Departments (N = 25)	Government (N = 14)	Research Institutes (N = 6)
Science & Practice	50.0	46.1	35.1	62.5
Promoting Health	46.6	44.5	38.6	65.6
Law, Policies & Ethics	50.7	50.7	40	66
One Health & Health Security	40.1	51.8	32.2	66
Leadership & System Thinking	44.0	62.9	40	68.1
Collaborations & Partnership	47.5	60.9	40.6	68.9
Communication, Culture & Advocacy	39.3	61.7	32.7	48.2
Governance & Resource Management	36.9	56.5	31	57.8
Professional Development & Reflective Ethical Practice	33.3	47.7	24.2	52.8
Organizational Literacy & Adaptability	38.8	56.5	45.6	58.3

HMO, Health Maintenance Organization; NGO, Non-Governmental Organization.

^a^ Response percent averages calculated for total items per category.

The project participants addressed concerns about the insufficient capability of the PH workforce in response to current and emerging PH threats and appropriately respond to at-risk groups using effective communication and implementation channels. In addition, the competency deficiencies appeared at all proficiency levels, including a limited capacity for innovation and difficulty in “thinking outside the box.” Participants commented on the importance to strengthen leadership competencies and trainings in system’s thinking [35].

Additionally, a cross-sectional, self-administered questionnaire survey of the Haifa School of Public Health alumni was performed with 127 MPH degree graduates. This was followed by 24 in-depth interviews with alumni from the same sample. The results demonstrated limited coordination and alignment between the academic curriculum and the jobs available for alumni, hindering better alumni professional development. Regular discussions, information sharing, and curriculum refinements between MPH program leaders and health sector leaders might address many of the concerns of the MPH degree graduates [33].

The triangulation of results from our multi-methods findings highlight the ongoing implementation gaps and the inadequate working relationships between community-based workers, clinical teams, and university academic leaders regarding their shared goals and knowledge. These “relational dynamics” are associated with a lack of frequent and timely communication between these key leaders, predicting the frustration heard by many in the study and the low quality and efficiency of the interactions between these key actors [42].

#### Academic PH Programs Maps

We mapped the Israeli PH curricula using the ASPHER List of Core Competences [37] to ensure HEIs effectively train PH professionals adequately to address current and future PH challenges. Representatives from five HEIs completed a survey of 57 competencies across six domains [38]. The competencies are covered in core and elective courses in undergraduate and MPH programs. Table 3 summarizes the coverage rates of competencies as reported by PH academic programs [38].

**TABLE 3:** Rates of competencies covered as reported by PH Academic programs for the SEEEPHI project, % of coverage (Israel, 2024).

Competency Domain	PH Academic Program	
	Undergraduate program (N = 1)	MPH graduate programs (N = 4)
Methods in Public Health	100	56–95
Socioeconomic Determinants of Health	100	75–98
Environmental Determinants of Health	36	40–100
Health Policy, Economics & Organization	60	40–100
Health Promotion and Prevention	91	40–90
Ethics	100	80–100

Our findings demonstrate that the Undergraduate Program addressed 79% of the total competencies, while the MPH graduate Programs addressed a range of 45%–84% of the total competencies, with considerable variability across the five institutions. In addition, since the master’s programs have different tracks and specializations and often their focus is different, we found that differences in training program structures can affect the comprehensiveness of competency coverage [38].

The analyses of phase 1 findings reveal several critical areas for action regarding the needed improvements in PH training programs in Israel:

1. Competency Coverage: As seen in Table 3, competency coverage distribution across different domains was uneven. Core areas such as Methods in PH and Socioeconomic Determinants are well-covered, while Environmental Determinants, Health Policy, Economics & Organization, and Health Promotion and Prevention are less addressed.
2. Practical Skills Deficiency: There were significant gaps between the competencies taught in training programs and those required in practice, particularly in Advocacy, Communication, Social Mobilization, Collaboration, Partnership, and Leadership.
3. Harmonization and Standardization: There is a need for greater harmonization of PH curricula content with international standards to ensure that graduates are adequately prepared for the workforce but at the same time allow for independence to determine different tracks according to the students’ preferences.
4. Innovative and Versatile Training: PH professionals should be equipped with versatile and innovative training to address current and emerging PH challenges effectively. In addition training competencies should be strengthened “on the job” with continuous education courses and employer initiatives to keep PH staff updated on current practices.

### Phase 2: Interventions

Based on analysis of phase 1 data, we developed and implemented the following three interventions:

1) An online interface using human-computer interaction (HCI) methods to enhance human-system collaboration.
2) A Leadership Academy for PH providing cutting-edge simulations and problem-based learning (PBL) training tools.
3) PH case studies using storytelling techniques to reach stakeholders and community engagement.

**FIGURE 1:**
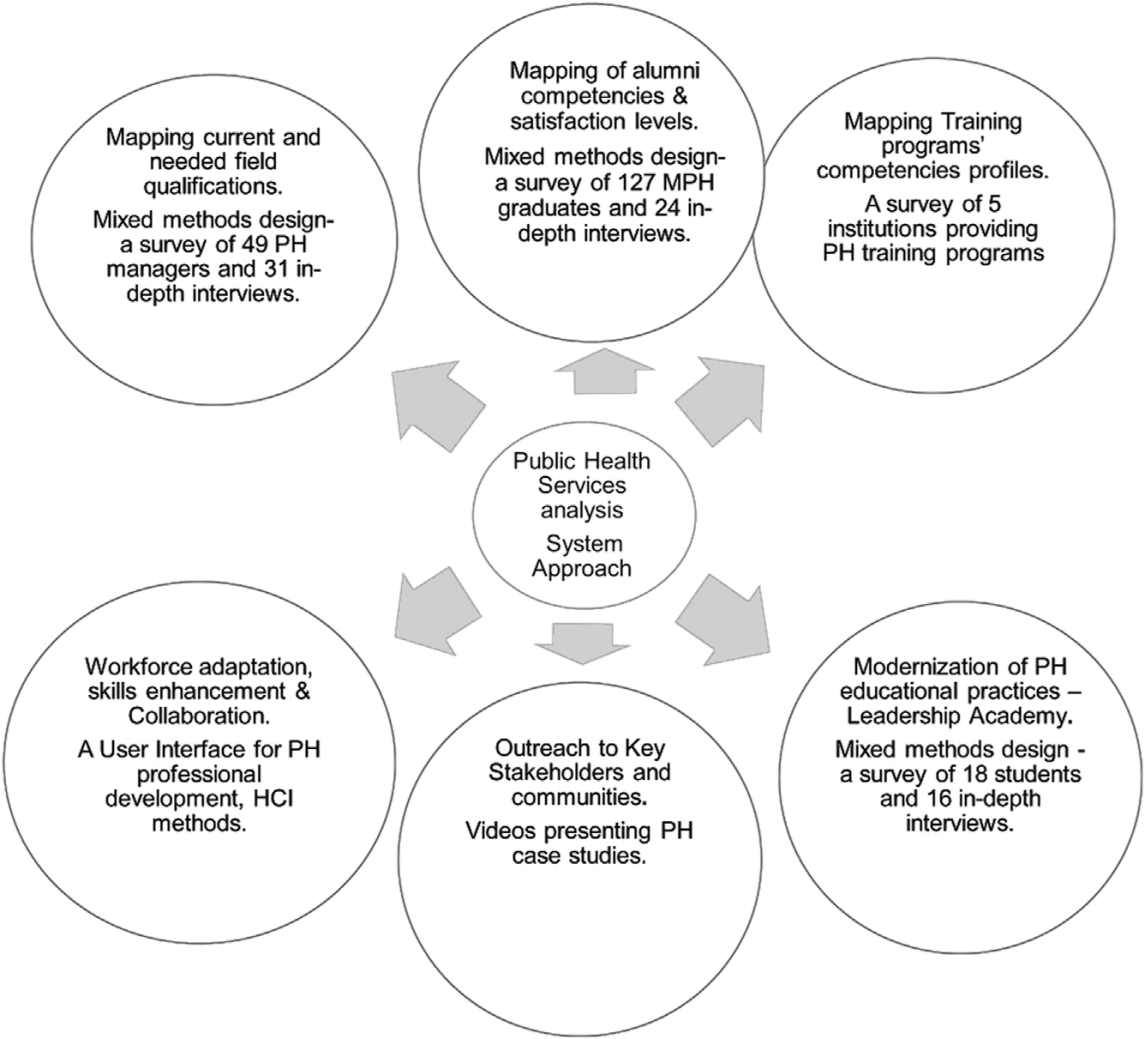
The SEEEPHI project logic model (Israel, 2024). SEEEPHI, Sharing European Educational Experience in Public Health for Israel; PH, public Health.

Figure 1 summarizes the SEEEPHI project logic model, including phases and tools.

The following sections describe the next stages of the project, including the interventions implemented for workforce adaptation, developing and building leadership capacity (assessed by survey number 4, followed by in-depth interviews), and promoting wide engagement by stakeholders. t.

#### Online Hub--Workforce Adaptation, Professional Development, and Collaboration

University public health educators aim to enhance collaboration between PH entities to promote professional development. Linking training and practice is often done by developing an innovative platform to support active participation in communication, data sharing, and collaboration. The online platform we developed aims to serve as a centralized hub for communication and collaboration between students, educators, employers, researchers, and PH organizations (preliminary version can be found at https://seeephi.eu/). The platform was developed using HCI working methods, including creating user scenarios to present personas, analyzing possible functionalities, and developing wireframes with mockups [43]. We used stakeholder participatory design methods for individual and institutional users and utilized the platform as an analytical tool for assessing the data in the system. This dynamic online platform will allow students to network with professionals and organizations in PH fields, which is crucial for career development and enhanced job opportunities [44]. Organizations can post internship and job opportunities, and students and others can apply directly. This direct connection with employers streamlines the hiring process and ensures that students and others are aware of available opportunities [45]. The online platform can track interactions, feedback, and outcomes, providing valuable data for continuous improvement. This data can help identify successful strategies and areas for future improvement, ensuring that the platform evolves to meet the PH system’s and its users’ needs.

#### Building Leadership Capacity

Based on the analysis conducted in phase 1, a training module to enhance PH students’ leadership and system thinking competencies was developed and implemented [32]. Built on the previous experiences developed by ASPHER using the ASPHER/WHO Europe Road Map for Professionalizing the Public Health Workforce [46], the module introduced students to the foundations of leadership in PH through a combination of key lectures, case study analysis using Problem-Based Learning (PBL) method, and immersive simulation training. The course deals with the salient leadership skills required for developing and improving teamwork within the PH system. The second part of the course is based on the problem-based learning (PBL) method and presents students with practical leadership challenges examined through case studies and practical simulations.

A complementary study explored the effectiveness of simulation-based training in developing leadership and decision-making skills in PH students [32]. 18 students from Ben Gurion University of the Negev participated in two different simulation scenarios. Their experiences and skill acquisition were compared to traditional face-to-face learning methods.

Four main themes emerged from the interviews: experiential learning effectiveness, differences between the two scenarios, simulation as a learning toolbox, and the relevance of simulation to professional training. The students reported improvements in their interpersonal communication skills [32]. The simulations provided realistic experiences of leadership, decision-making, and teamwork challenges [47]. Participants appreciated the simulations’ interactive and practical nature in developing effective leadership skills, which helped them apply theoretical knowledge in real-world contexts to serve society best [32].

#### Stakeholder Engagement

We designed the engagement intervention aimed at building partnerships with key professional groups needed to promote the integration of PH graduates across the broader PH workforce. This encouraged awareness for prioritization of resource allocation for PH and in advancing professional recognition of the Israeli PH workforce.

This stage focused on outreach to stakeholders through an increased awareness campaign on the roles PH professionals play entitled “This Is Public Health (TIPH)” campaign [48]. The ongoing TIPH campaign uses innovative storytelling techniques to develop a set of short videos presenting PH case studies (https://www.aspher.org/this-is-public-health-tiph.html).

The videos were presented by PH professionals and students and distributed on different social media platforms (e.g., Facebook, Twitter, etc.) and PH conferences. (https://www.tiktok.com/@publichealthil?_t=8mmiImtsmjh&_r=1).

Innovative storytelling can simplify complex information, making it easier for stakeholders to understand and support PH leadership. This is crucial in PH, where clear communication can influence behaviors and policy decisions [49]. The platform helps to inspire significantly more engagement and commitment, build trust and credibility by connecting with stakeholders, and sharing PH’s personal stories and real-life examples of PH messaging. Stakeholders are more likely to trust information from relatable and authentic sources [50]. This approach led to better understanding, acceptance, and action among stakeholders, ultimately improving PH outcomes.

**FIGURE 2:**
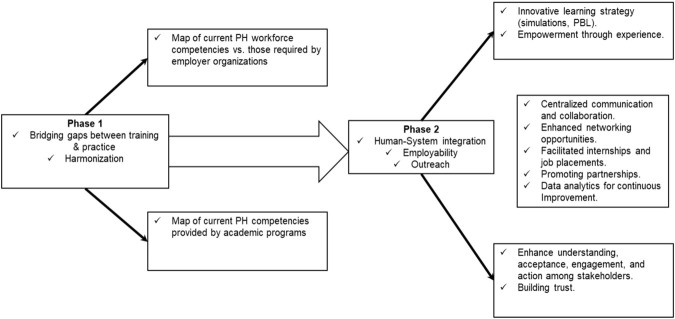
Project SEEEPHI key outputs (Israel, 2024).

The key outputs of the SEEEPHI project phases are summarized in Figure 2.

## Discussion

We describe the integrated findings of a multi- method and -level, multi jurisdictional, 3-year SEEEPHI project, which applied a whole system’s learning approach to analyze and strengthen PH system in Israel. The novel SEEEPHI research project utilized a rich diversity of system-based and participatory-based human-centered design tools to analyze and improve the PH workforce training system by applying a learning organization approach.

The PH system is complex and multifaceted and requires a holistic and integrated approach to address challenges efficiently. The interconnectedness of various components within the PH ecosystem means that changes in one part can have ripple effects throughout the entire system [51]. Re-designing the PH system to be a learning-adaptive system and recognizing the need for a complexity-informed paradigm shift may encourage practitioners to consider the broader context and interdependencies rather than focusing solely on individual competencies, one-off issues or isolated interventions [52].

**FIGURE 3:**
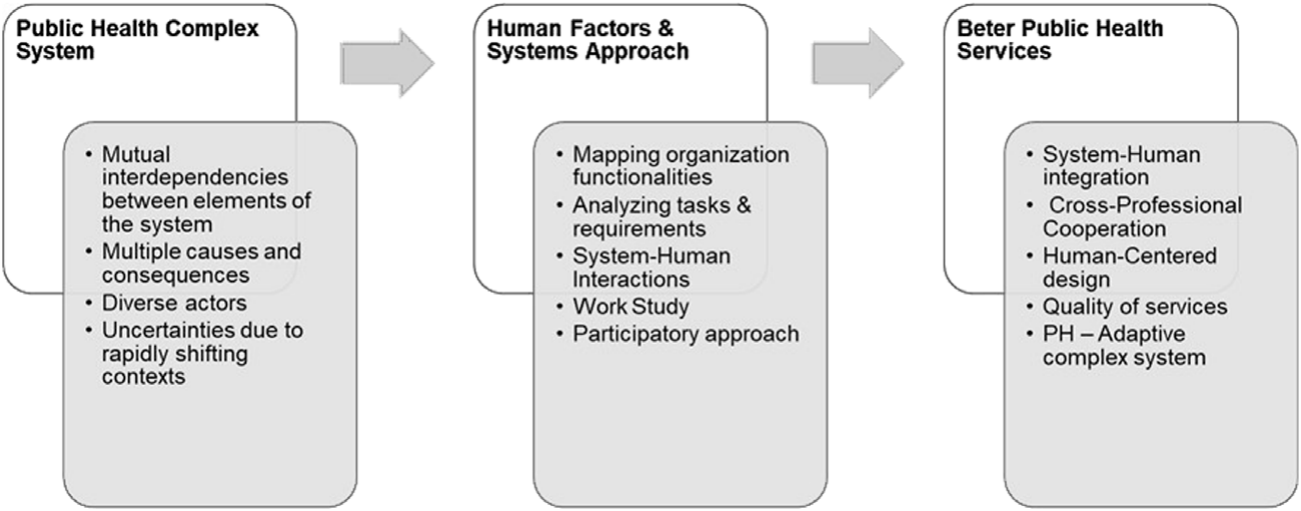
A human factors system-based pedagogical approach to Public Health curriculum design (Israel, 2024).

Applying a human factors approach to design evidence driven interventions enabled the SEEEPHI project members to characterize the interlinks of the PH system and to recognize that the individuals within the PH system - the PH workforce, employers, academic staff, policymakers, and stakeholders–all play a crucial role in shaping the system’s dynamics and outcomes [53]. Acknowledging the many human factors that influence how people within the PH system perceive, process, and respond to the work environment, interventions, and initiatives can help facilitate the development, implementation, and adoption of more user-centric, and effective change strategies in design, implementation, and oversight of PH services [54]. The tools and methods SEEEPHI deployed can offer valuable insights and strategies for optimizing the performance and outcomes of PH education and services. Figure 3 describes the processes and outcomes of applying human factors and the system’s approach methods to accelerating PH improvements to benefit society.

The PH system has long been viewed through a traditional approach. However, as the landscape of PH challenges becomes increasingly complex and wicked, there is a growing recognition that this approach is no longer sufficient or sustainable [55]. PH systems must continually evolve in response to internal and external pressures, such as disasters, emerging diseases, demographic shifts, and policy changes. Understanding this complex adaptive nature of the PH system can enhance resilience and the capacity to respond to new challenges. Embracing a system’s approach using dynamic systems mapping can provide a comprehensive understanding of the PH system, identify leverage points for improvement, and facilitate continuous learning to strengthen the workforce and more effectively address complex PH challenges [56].

### Limitations

While the results of SEEEPHI are promising, the project had several limitations that should be considered in interpreting its findings. First, it was conducted solely in the Israeli PH context, which may limit the generalizability of the findings to other countries with different PH and social policy systems, educational schemes and challenges. Second, although the project involved various stakeholders, the sample sizes for some groups were relatively small, potentially not capturing the full range of perspectives within the PH workforce and limiting the study’s external generalizability. Additionally, self-reported data in surveys and interviews may be subject to a response bias affecting interpretation. Third, the interventions we implemented, such as the leadership training academy and online collaboration platform, were recently piloted, and their long-term effects remain to be evaluated. Finally, while the systems approach provides valuable insights, fully capturing and addressing the complexities of the PH system remains challenging.

Future research should aim to expand the geographical scope, increase sample sizes, conduct longitudinal evaluations of interventions, and refine methods for analyzing complex PH adaptive systems.

### Conclusion

We need a growing and competent public health workforce to address and improve society’s resilience given ongoing threats and day-to-day PH activities. Incorporating a systems-based human factors approach enables more integrated and sustainable health solutions and diplomacy to improve PH programs’ usability, acceptance, and impact. A systems approach encourages the use of diverse methods, and interdisciplinary and interprofessional collaboration to develop a more comprehensive understanding of the PH system’s unique challenges and create more robust interventions [57]. A more nuanced understanding is needed in appreciating that the system is not merely the sum of its parts, but the result of its dynamic interactions. This can result in more innovative, effective, and sustained solutions [58].

PH services and higher education program leaders should work more collaboratively to re-evaluate PH curricula to better align with the required knowledge, skills, and competencies and ensure they are responsive to the dynamic and multifaceted nature of real-world PH challenges. Developing new learning skills, fostering innovative thinking, and improving self-learning and problem-solving skills will better prepare graduates to meet the evolving needs of the profession and society resilience.
